# Development of synthetic biology tools to engineer *Pichia pastoris* as a chassis for the production of natural products

**DOI:** 10.1016/j.synbio.2021.04.005

**Published:** 2021-05-03

**Authors:** Jucan Gao, Lihong Jiang, Jiazhang Lian

**Affiliations:** a Key Laboratory of Biomass Chemical Engineering of Ministry of Education, College of Chemical and Biological Engineering, Zhejiang University, Hangzhou, 310027, China; b Hangzhou Global Scientific and Technological Innovation Center, Zhejiang University, Hangzhou, 310027, China

**Keywords:** *Pichia pastoris*, Natural products, Synthetic biology, CRISPR/Cas9, Heterologous gene expression

## Abstract

The methylotrophic yeast *Pichia pastoris* (a.k.a. *Komagataella phaffii*) is one of the most commonly used hosts for industrial production of recombinant proteins. As a non-conventional yeast, *P. pastoris* has unique biological characteristics and its expression system has been well developed. With the advances in synthetic biology, more efforts have been devoted to developing *P. pastoris* into a chassis for the production of various high-value compounds, such as natural products. This review begins with the introduction of synthetic biology tools for the engineering of *P. pastoris*, including vectors, promoters, and terminators for heterologous gene expression as well as Clustered Regularly Interspaced Short Palindromic Repeats/CRISPR-associated System (CRISPR/Cas) for genome editing. This review is then followed by examples of the production of value-added natural products in metabolically engineered *P. pastoris* strains. Finally, challenges and outlooks in developing *P. pastoris* as a synthetic biology chassis are prospected.

## 1 Introduction

Natural products generally refer to secondary metabolites isolated from animals, plants, and microorganisms, which have special physiological activities. Many high-value natural products play an extremely important role in medicine, cosmetics, and industry [1]. However, natural products generally have complex chemical structures and are extracted from sources with long growth periods and extremely low abundance, resulting in short supply and high cost, particularly those with plant origins [2]. With the development of synthetic biology, various microorganisms including *Pichia pastoris* (a.k.a. *Komagataella phaffii*) have been developed as cell factories to produce natural products [[3], [4], [5]]. Compared with bacterial cell factories (i.e. *Escherichia coli*), the ability of post-translational modifications and the presence of inner membrane systems make yeasts including *P. pastoris* preferred hosts to express eukaryotic complex proteins, such as cytochrome P450s (CYPs), which are often involved in the biosynthesis of natural products [6]. When compared with the model yeast *Saccharomyces cerevisiae*, *P. pastoris* has the advantage of strong and tightly regulated promoters for high level expression of recombinant proteins [7]. For example, the expression level of the target gene can account for more than 30% of the total proteins of *P. pastoris*, which is much higher than that in *S. cerevisiae* [8]. In addition, hyperglycosylation is another concern for expressing eukaryotic proteins in the *S. cerevisiae* expression system [9]. Natural product biosynthetic pathway enzymes (i.e. polyketide synthases and CYPs) are generally found to have relatively low catalytic activities, which should be expressed at high levels to achieve efficient biosynthesis. Therefore, *P. pastoris* is a promising host for large-scale production of natural products, particularly those with eukaryotic origins.

**Fig. 1:**
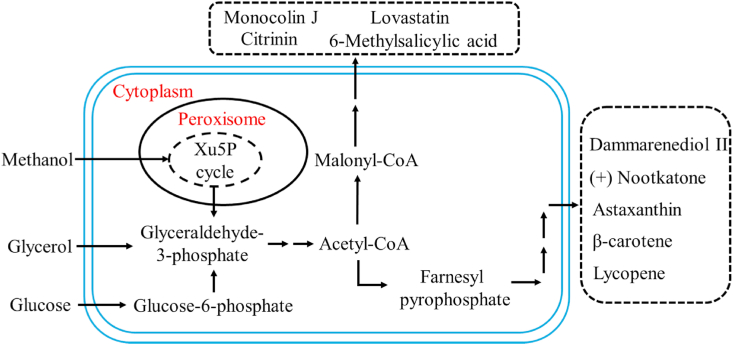
*P. pastoris* as a cell factory for the production of natural products. *P. pastoris* converts various carbon sources (i.e. methanol, glycerol, and glucose) into a few central metabolites (i.e. pyruvate and acetyl-CoA), which serve as the precursors for the biosynthesis of a variety of natural products (i.e. terpenoids, polyketides, and flavonoids).

Although generally considered as a non-conventional yeast, genetics, physiology, and cell biology of *P. pastoris* have been studied in-depth [4]. The genomes of *P. pastoris* GS115 and CBS7435 have been sequenced and annotated, and genome-scale metabolic models have been constructed by analyzing the metabolic patterns [10]. Metabolomics studies indicate that the intermediate metabolites of *P. pastoris* and *S. cerevisiae* are very similar, with identical metabolites up to 90% [11]. The exploration of the genetic background of *P. pastoris* has laid a solid foundation for customized modification of *P. pastoris*. Currently, besides therapeutic proteins and enzymes [12], *P. pastoris* has been engineered to produce various chemicals and value-added compounds, such as d-lactic acid [13], 2,3-butanediol (BDO) [14], 2-phenylethanol [15], isobutanol and isobutyl acetate [16], carotenoids [17], lovastatin [18], and nootkatone [19] (Fig. 1).

In this review, the synthetic biology tools needed for the construction and optimization of natural product biosynthetic pathways in *P. pastoris* are firstly summarized, in particular the heterologous gene expression system and the CRISPR/Cas (Clustered Regularly Interspaced Short Palindromic Repeats/CRISPR-associated System) based genome editing system. Subsequently, several examples of the establishment of *P. pastoris* as cell factories for the production of terpenoids, polyketides, and flavonoids are introduced. Finally, future perspectives in the development of novel synthetic biology tools for the assembly and integration of multi-gene biosynthetic pathways and high throughput genome engineering are discussed.

## 2 Synthetic biology toolkit for *P. pastoris*

### 2.1 Gene expression vectors

The most common way to introduce exogenous genes into *P. pastoris* is to construct recombinant vectors. The method of plasmid maintenance in yeast is through auxotrophic markers or resistance selection markers (Table 1) [20]. Plasmids can be divided into episomal plasmids and integrative plasmids according to the way existing in the host. Unfortunately, the episomal plasmids suffer from low stability and genome integration is generally preferred for high level expression of heterologous genes in *P. pastoris*. Usually the vector is linearized and integrated into the *P. pastoris* genome in a single copy manner. For example, the vectors pPIC9K-His and pHIL-S1 can be linearized by *Sal*I for the integration into the *HIS4* locus of *P. pastoris* GS115, which are then screened in histidine-dropout medium to obtain single-copy integrated strains [21,22]. In addition to single-copy integration, multi-copy integration is generally demanded for high-level expression of the target proteins. The pPIC series of vectors are commonly used integrative vectors in *P. pastoris* [[23], [24], [25], [26]], which enable the screening of the multi-copy integration strains under high concentration of antibiotics, a mechanism known as post-transformation amplification [[27], [28], [29], [30]]. In addition to the formation of tandem repeats via post-transformation amplification, multi-copy strains can be constructed by integrating into the repetitive sequences of the *P. pastoris* genome, such as the ribosomal DNA (rDNA) sequences [13].

**Table 1:** Gene expression vectors commonly used in *P. pastoris*.

Plasmid backbone	ARS or integration locus	Copy numbers	Selection markers	Episomal or Integrative	Reference
pHIL-S1^a^	*HIS4*	1.0	*HIS4*	Integrative	[21]
pPIC9K^a^^,^^b^ or pPIC9K-His^a^^,^^b^	*HIS4*/*AOX1*	1.0 or 4.0–6.0	*HIS4*/G418^r^	Integrative	[23,[34], [35], [36], [37]]
pPIC3.5K^b^	*HIS4*/*AOX1*	1.0	*HIS4*/G418^r^	Integrative	[24]
pGAPZ^b^^,^^c^	*GAP*	1.0 or 21	Zeocin^r^	Integrative	[14,27,38]
pPICZ	*AOX1*	1.0 or 10.0	Zeocin^r^	Integrative	[25,26,28]
pAO815	*HIS4*/*AOX1*	1.0	*HIS4*	Integrative	[39]
pPink HC or pPink LC	*TRP2*	2.0–4.0	*ADE2*	Integrative	[40,41]
pGLY2664^b^	*TRP2*	2.0–12.0	*ADE2*/Zeocin^r^	Integrative	[42]
pUC19^c^	rDNA	~9.0	Zeocin^r^	Integrative	[13]
pPICZα^a^^,^^b^	*AOX1*	~10.0	Zeocin^r^	Integrative	[29]
pMCO^a^	*HIS4*	2.0–16.0	G418^r^	Integrative	[30]
pGHYB	*GAP*	~3.0	Hygromycin^r^	Integrative	[43]
pPIC9K^d^	panARS	~18.0	*HIS4*	Episomal	[32]
pPIC9K^d^	PARS1	~15.0	*HIS4*	Episomal	[32]
pPIC9K^d^	mitoARS	~14.0	*HIS4*	Episomal	[32]
pPIC9K^d^	PpARS2	~4.0	*HIS4*	Episomal	[32]
pPIC9K^d^	ScARS	~4.0	*HIS4*	Episomal	[32]
pSEC-SUMO	panARS	~19.0	Zeocin^r^	Episomal	[33]
pMito	mtDNA	~3.0	*HIS4*	Episomal	[44]
pPICZαBHF^a^	PARS1	~7.0	Zeocin^r^	Episomal	[45]
pBGP1^a^	PARS1	N.A.	Zeocin^r^	Episomal	[46,47]
pPEHα^a^	PARS1	N.A.	*HIS4*	Episomal	[48]

Note.

a These plasmids contain a signal peptide for protein secretion.

b These *P. pastoris* integrative plasmids have both auxotrophic markers (i.e. *HIS4* and *ADE2*) for single-copy integration and resistance markers (i.e. G418^r^ and Zeocin^r^) for multi-copy integration. High-copy strains can be generally constructed by screening on high concentration of antibiotics.

c High-copy strains (up to 21 copies) were constructed by integrating the expression cassettes into the ribosomal DNA loci and screening under high Zeocin concentration conditions. Insert the *HIS4* gene into the plasmid to obtain a single copy strain.

d With pPIC9K as the backbone, a series of episomal plasmids were constructed by adding different yeast replicons (ARSs).

Nevertheless, episomal expression possesses unique advantages for several applications, such as the combinatorial optimization of multi-gene biosynthetic pathways and the development of efficient CRISPR-based genome editing tools [31]. In this case, a set of episomal vectors with various autonomously replicating sequences (ARSs) have been constructed and systematically compared for their transformation efficiency, copy numbers, and reproductive stability (Table 1) [32]. Of a particular note, panARS, a broad host ARS derived from *Kluyveromyces lactis*, was found to enable the highest plasmid stability and chosen for the development of an efficient CRISPR/Cas9 system for *P. pastoris* [33].

### 2.2 Promoters and terminators

Promoters are considered as the most important synthetic biology elements and have direct impacts on the expression of the transcription units. The selection of appropriate promoters with the desirable strength is essential to construct well-controlled synthetic biology modules and to achieve optimal expression of the target genes. The alcohol oxidase 1 promoter (*pAOX1*) and the glyceraldehyde 3-phosphate dehydrogenase promoter (*pGAP*) are two most commonly used promoters [49]. The *AOX1* promoter is generally considered as the strongest promoter of *P. pastoris*, which is strongly induced by methanol and inhibited by glycerol, ethanol, and glucose. Under the full induction conditions, Aox1p accounted for more than 30% of the total cellular proteins (Table 2) [50]. The *GAP* promoter is a strong constitutive promoter, whose expression strength is relatively stable. The expression level of some foreign proteins under the control of *pGAP* can reach up to the level of g/L [51].

**Table 2:** *P. pastoris* promoters commonly used for the expression of heterologous genes.

Promoter	Type	Relative Strength^a^		Locus tag	Reference
		Methanol	Glucose		
*pAOX1*	Methanol inducible	~18	/	*PAS_chr4_0821*	[49]
*pAOX176*^b^	Methanol inducible	~26	/	/	[55]
*pAOX737-△D+3D*^c^	Methanol inducible	~28	/	/	[56]
*pAOX2*	Methanol inducible	1.8 ± 0.2	/	*PAS_chr4_0152*	[52]
*pAOX2-*mutant^d^	Methanol inducible	~18	/	/	[58]
*pCAT1*	Methanol inducible	~18	/	*PAS_chr2-2_0131*	[62]
*pCAT1-*mutant^e^	Methanol inducible	~23	/	*PAS_chr2-2_0131*	[62]
*pDAS2*	Methanol inducible	24.1 ± 3.3	/	*PAS_chr3_0834*	[52]
*pFLD1*	Methanol inducible	10.9 ± 1.1	/	*PAS_chr3_1028*	[52]
*pPMP20*	Methanol inducible	16.4 ± 3.3	/	*PAS_chr1-4_0547*	[52]
*pDAS1*	Methanol inducible	14.7 ± 2.7	/	*PAS_chr3_0832*	[52]
*pFDH1*	Methanol inducible	14.2 ± 2.2	/	*PAS_chr3_0932*	[52]
*p0374*	Methanol inducible	~4	/	*PAS_chr3_0374*	[49]
*p0319*	Methanol inducible	~3	/	*PAS_chr1-1_0319*	[49]
*p0547*	Methanol inducible	~2	/	*PAS_chr1-4_0547*	[49]
*p0472*^f^	Constitutive	~3 or 22	~0.5	*PAS_chr2-1_0472*	[49,54]
*pGAP*	Constitutive	1.0	~2.3	*PAS_chr2-1_0437*	[49]
*p0769*	Constitutive	~0.4	~1.8	*PAS_chr2-1_0769*	[49]
*p0072*	Constitutive	~0.3	~1.4	*PAS_chr1-1_0072*	[49]
*pPDC*^g^	Constitutive	~0.5	~3.5 or 4.7	*PAS_chr3_0188*	[49,53]
*pPDI1*	Constitutive	~0.5	~0.4	*PAS_chr4_0844*	[63]

Note.

a Promoter strengths are normalized to that of the constitutive promoter (*pGAP*) under methanol conditions.

b *pAOX176* is generated by removing a small number of bases before the TATA box of *pAOX1*.

c *pAOX737* represents that the bases from −940 to −737 bp of *pAOX1* are deleted. Region D is defined as the position of −638 to −510 bp in *pAOX1*. -△D+3D means the deletion of region D followed by the addition of 3 copies of region D at the 5′-end of *pAOX1*.

d *pAOX2*-mutant is obtained by mutating the bases at −255 and −456 positions of *pAOX2*.

e *pCAT1*-mutant represents that the bases from −433 to −411 bp of *pCAT1* are duplicated.

f *p0472* is 3 times stronger than *pGAP* when expressing recombinant α-amylase, while 22 times stronger when expressing xylanase.

g *pPDC* is 1.5 times stronger than *pGAP* when expressing recombinant α-amylase, while 2 times stronger when expressing human growth hormone.

To explore metabolic engineering and synthetic biology applications, a series of promoters with different properties have been characterized and engineered [52]. Based on RNA-seq results, *p0188* (Pyruvate decarboxylase isozyme, locus tag: *PAS_chr3_0188*) was determined to be the strongest constitutive promoter and Aslan et al. found that *p0*188 had a strong driving force and the strength could reach up to 2-fold as that of *pGAP* [53]. By combining RNA-seq and mRNA folding free energy, 16 promoter candidates were selected and characterized with glucose, glycerol, or methanol as the sole carbon source, respectively. The *p0547* (peroxidase promoter, locus tag: *PAS_chr1-4_0547*) and *p0472* (mitochondrial alcohol dehydrogenase isozyme III, locus tag: *PAS_chr2-1_0472*) were determined to be the strongest methanol‐inducible and constitutive promoters to express *α*‐amylase, respectively [49]. Interestingly, in another separate study, Karaoglan et al. found that the *ADH3* promoter (locus tag: *PAS_chr2-1_0472*) performed even better than the *AOX1* promoter when using methanol as the sole carbon source to express *Aspergillus niger* xylanase [54].

In addition to the mining and characterization of endogenous promoters, existing promoters can be modified to possess the desirable features. For example, at least 12 cis-acting elements were found in the *AOX1* promoter and a promoter library of *pAOX1* is constructed by deleting and replicating the putative transcription factor binding modules. The activity range of the promoter library is 10%–160% of the wild-type *pAOX1* [55]. A key transcription factor, called Mxr1p, was determined to be essential for the induction of *pAOX1* by methanol [56]. Due to the toxicity of methanol, Shen et al. established a methanol-free expression system using *AOX1* promoter mutants, in which the mutant promoter was induced by dihydroxyacetone and suppressed by glucose. The strength of this protein expression system can reach 50%–60% of the wild-type *pAOX1* expression system [57]. Dai et al. identified a *pAOX2* mutant, whose expression level was even higher than that of *pAOX1*. DNA sequencing revealed two point mutations at positions of −529 bp and −255 bp responsible for the dramatically improved promoter strength [58].

Terminators have been found to have important regulatory effects on transcription termination and the half-life of mRNA in *S. cerevisiae* [59,60]. However, the significance of terminators is largely overlooked and little work has been done on the characterization of *P. pastoris* terminators. Vogl et al. tested the effect of different terminators on the expression of eGFP (enhanced green fluorescent reporter protein) under the control of *AOX1* promoter and *AOX1* terminator was found to enable the highest fluorescence intensity. In addition, inserting *Not*I restriction site into the *AOX1* terminator can further increase the fluorescence intensity by 37% [52]. Ito et al. characterized 72 terminators derived from *P. pastoris*, *S. cerevisiae*, and synthetic terminators, and found that the tunable range could reach up to 17-fold. Interestingly, the *S. cerevisiae* terminators could maintain their function after being transferred to *P. pastoris* [61]. These preliminary studies indicated the significance of terminators in regulating the expression level of heterologous genes and more mechanistic studies should be carried out in the near future.

### 2.3 Genome editing tools

As a fundamental tool, genome editing technology is essential for establishing *P. pastoris* as cell factories for recombinant proteins and value-added compounds. In the very beginning, site-directed gene integration and gene knockout were achieved through homologous recombination. Construction of a selection marker-containing plasmid that is capable of gene replacement in *P. pastoris* is one of the first genome editing tools [64]. For example, *HIS4*, *URA3,* and *URA5* genes are often used as selection markers in the corresponding defective *P. pastoris* strains [65]. However, these genome editing techniques usually leave selection marker expression cassettes in the host, which is not desirable for subsequent genetic manipulations and industrial applications. To enable multiple rounds of genome editing, Cre/*lox*P system was introduced into *P. pastoris*. Cre is a site-specific recombinase that specifically recognizes and recombines genes between two *lox*P loci. The advantage of this technology is that antibiotic resistance genes can be used for screening first and then recycled after the disruption of the target gene [66]. In addition, *mazF*, a toxic gene from *E*. *coli*, was used to construct a set of counter-selection techniques for marker-less genome editing in *P. pastoris* [67].

**Fig. 2:**
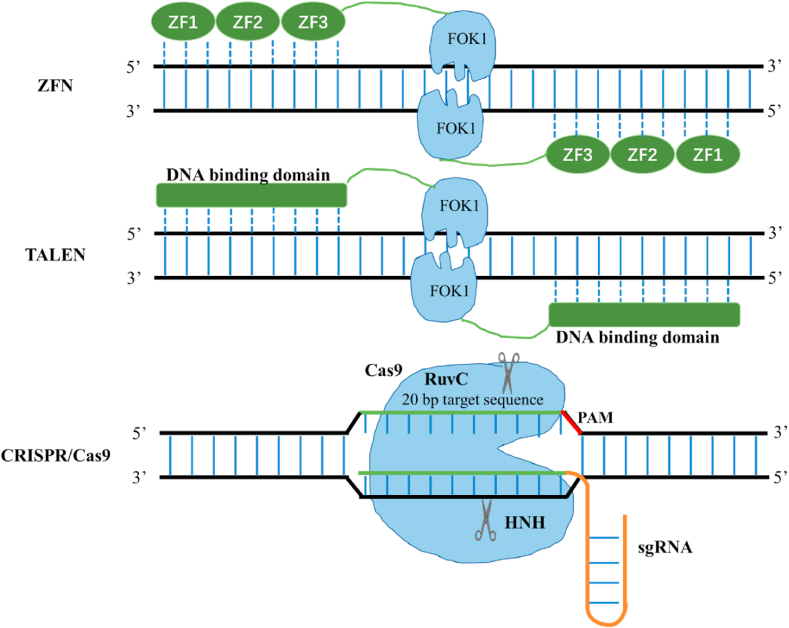
Three emerging genome editing techniques, including ZFN, TALEN and CRISPR/Cas9.

In recent years, emerging genome editing tools, such as ZFN (Zinc-finger nucleases), TALEN (transcription activator-like effector nucleases), and CRISPR/Cas, have revolutionized our capability of genetic manipulations of microbial cell factories (Fig. 2). These technologies use specific nucleases to create double-strand breaks (DSB) at the corresponding loci, which are repaired by homologous recombination (HR) or non-homologous end joining (NHEJ) to achieve the desirable genome editing. Particularly, the CRISPR/Cas system is the most widely used and most powerful genome editing technology. The CRISPR/Cas9 system is derived from the immune defense systems of bacteria and archaea [68], and has received in-depth research in microbial cell factories development, plant breeding, animal breeding, disease modeling, and biotherapy [69]. Weninger et al. systematically optimized the CRISPR/Cas9 expression system to achieve efficient and precise genome editing in *P. pastoris*, including but not limited to Cas9 coding sequences, gRNA sequences, gRNA structures (i.e. with ribozyme sequences), and promoters for the expression of Cas9 and gRNAs [70]. Among 95 combinations, only 6 constructs were found to be functional for genome editing, indicating the necessity for further optimization (Table 3). For example, Gu et al. found that the replacement of the origin of replication of the bearing plasmid from PARS1 to panARS increased the disruption efficiency of *ADE2* locus from ~10% to ~80% [32]. In addition, Dalvie et al. developed a sequencing-based strategy for the design of host-specific cassettes for modular and efficient expression of gRNAs and achieved high genome editing efficiency up to 95% [71]. Besides gene disruption, multiplex integration of heterologous genes is another essential synthetic biology tool for establishing *P. pastoris* as cell factories for natural products. Simultaneous integration of multiple genes was reported in a *KU70*-deficient *P. pastoris* strain, with an integration efficiency ranged from 57.7% to 70% and 12.5%–32.1% for double- and triple-loci, respectively [72].

**Table 3:** CRISPR/Cas9 systems for genome editing of *P. pastoris*.

Cas9 promoter	sgRNA promoter, promoter type	Host	Target(s)	Donor length	Efficiency	References
*pHTX1*	*pHTX1*, II	CBS7435	*GUT1*	1000 bp	87–94%	[70]
*pENO1*	*ptRNA-tRNA1*, III	NRRL Y-11430	*GUT1*	500 bp	95%	[71,73]
*pHTX1*	*pHTX1*, II	GS115 △*ku70*	2 loci^a^	1000 bp	57.7–70%	[72]
*pHTX1*	*pHTX1*, II	GS115 △*ku70*	3 loci^b^	1000 bp	12.5–32%	[72]
*pGAP*	*pHTX1*, II	GS115	*MXR1*	~600 bp	>80%	[74]
*pGAP*	*pSER*, III	GS115	*ADE2*	250 bp	80%	[32]
*pGAP*	*pHTX1*, II	GS115	*Gt1*	None	100%	[31]
*pHTX1*	*pHTX1*, II	CBS7435 △*ku70*	*GUT1*	1000 bp	78–91%^c^	[75]
*pHTX1*	*pHTX1*, II	CBS7435 △*ku70*	*GUT1*	1000 bp	80–95%^d^	[75]
*pHTX1*	*pHTX1*, II	CBS7435 △*ku70*	*GUT1*	1000 bp	100%^e^	[75]
*pHTX1*	*pHTX1*, II	KM71	*PDC1*	1000 bp	N.A	[76]

a Any two loci of *pAOX1*, *pFLD1,* and *pTEF1* were simultaneously targeted.

b *pAOX1*, *pFLD1,* and *pTEF1* were simultaneously targeted. None means that no donor was added and DSB was repaired by NHEJ during CRISPR editing.

c Integration efficiency using the canonically designed DNA donor.

d Integration efficiency using DNA donor with a selection marker (zeocin^r^).

e Integration efficiency using DNA donor with a replicon (ARS).

## 3 Engineering of *P. pastoris* to produce natural products

### 3.1 Terpenoids

Terpenoids are value-added natural products derived from mevalonate and widely existed in nature, including but not limited to higher plants, fungi, and microorganisms. Many terpenoids have been found applications in medicine, food, cosmetics, animal feeds, and industry, leading to the exploration of the production of terpenoids using microbial cell factories. Bhataya et al. introduced the lycopene biosynthetic pathway into non-carotenogenic *P. pastoris* for the first time. Two lycopene-pathway plasmids were constructed, with plasmid pGAPZB-EBI* harboring genes *crtE*, *crtB,* and *crtI* and plasmid pGAPZB-EpBpI*p harboring the same set of genes with a peroxisomal targeting sequence (PTS1). Similar amount of lycopene was produced in the two yeast strains, indicating that the supply of FPP might be limited in *P. pastoris*. One clone expressing pGAPZB-EpBpI*p with the highest lycopene production was identified and further optimized by investigating the effects of culturing conditions (i.e. carbon sources and aerations). Finally, the production of lycopene reached up to 73.9 mg/L in the basic medium with glucose as the carbon source [77]. Later, β-carotene was synthesized by additionally integrating the lycopene β-cyclase gene from *Ficus carica* into the chromosome of the lycopene-producing strain, leading to the production of 339 μg of *β*-carotene per gram dry cell weight (DCW) [17]. Starting from the β-carotene-producing strain, further introduction of β-carotene ketolase gene (*crtW*) and β-carotene hydroxylase gene (*crtZ*) from *Agrobacterium aurantiacum* resulted in the production of 3.7 μg/g DCW of astaxanthin in *P. pastoris* [78]. In another study, Vogl et al. characterized a panel of promoters in the methanol utilization pathway of *P. pastoris*, which were further employed for combinatorial optimization of the β-carotene biosynthetic pathway. With different combinations of the methanol inducible promoters, the production of β-carotene can be varied for more than 10-fold. Via choosing appropriate promoters from the established promoter library, the yield of β-carotene reached up to 5 mg/g DCW [52].

(+)-Nootkatone, an excellent fragrance and insect repellent, have also been successfully produced in *P. pastoris*. The introduction of valencene synthase resulted in the biosynthesis of (+)-valencene. Followed by the co-expression of the premnaspirodiene oxygenase from *Hyoscyamus muticus* (HPO) and the cytochrome P450 reductase from *Arabidopsis thaliana*, (+)-valencene was hydroxylated to produce trans-nootkatol. Trans-nootkatol was then oxidized to (+)-nootkatone by the intrinsic activity of *P. pastoris*. The production of (+)-nootkatone was 17 mg/L in a shake flask and 208 mg/L in a bioreactor, respectively [19]. Interestingly, the overexpression of *RAD52*, which is responsible for DNA repair and recombination, improved the production of trans-nootkatol by 5-fold [79].

**Fig. 3:**
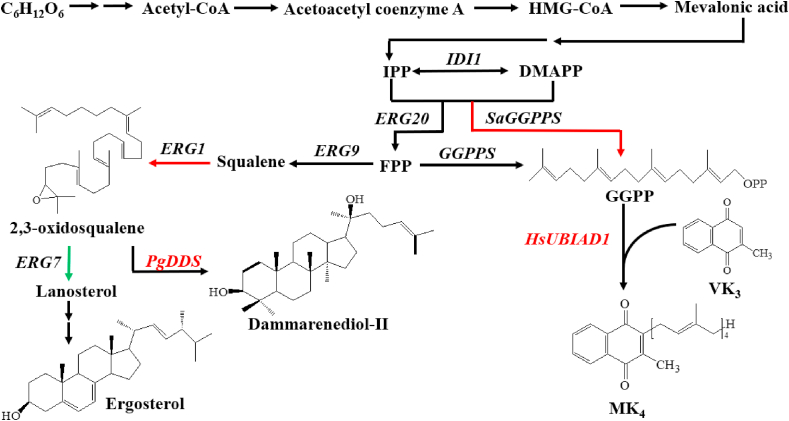
Biosynthetic pathway of dammarenediol-II and MK_4_ in *P. pastoris*. This study was carried out on the basis of the natural triterpene synthesis pathway. By introducing the exogenous *PgDDS* gene, encoding a dammarenediol synthase, the target compound dammarenediol-II was produced from 2,3-oxidosqualene in *P. pastoris*. The green arrow indicated the down-regulated gene (*ERG7*) and the red arrow indicated the overexpressed genes. (For interpretation of the references to colour in this figure legend, the reader is referred to the Web version of this article.)

Dammarenediol-II is a triterpenoid with multiple pharmacological activities. On the basis of the natural triterpene biosynthesis pathway [80,81], Liu et al. introduced *PgDDS* from *Panax ginseng*, encoding a dammarenediol-II synthase that catalyzed the production of dammarenediol-II from 2,3-oxidosqualene, to successfully construct a dammarenediol-II producing *P. pastoris* strain (Fig. 3). By increasing the expression of *ERG1* to enhance the supply of 2,3-oxidosqualene and downregulating the expression of *ERG7* to decrease the production of lanosterol from 2,3-oxidosqualene, the yield of dammarenediol-II was increased from 0.03 mg/g DCW to 0.736 mg/g DCW. Finally, by extra supplementation of 0.5 g/L squalene into the culture medium, the yield of dammarenediol-II reached up to 1.073 mg/g DCW.

Similarly, Sun et al. established a menaquinone-4 (MK-4) *P. pastoris* cell factory by introducing a heterologous gene encoding *Homo sapiens UBIAD1* (*HsUBIAD1*), which can produce MK-4 from phylloquinone (VK1) or menadione (VK3). *HsUBIAD1* was cloned into pGAPZA (with the constitutive promoter *pGAP*) and pPICZA (with the inducible promoter *pAOX1*) and the effect of promoters on the expression of the target gene was investigated. It was found that the vector pGAPZA (with the target gene *HsUBIAD1* under the control of *pGAP*) resulted in higher protein expression level. Then the geranylgeranyl pyrophosphate synthase gene (*GGPPS*) from *Sulfolobus acidocaldarius* was fused with the endogenous isopentenyl diphosphate isomerase gene (*IDI1*), and the resultant *IDI1*-*GGPPS* chimeric gene was integrated into the 28S ribosomal DNA (rDNA) loci in a multi-copy manner using a modified integrative vector (pGrG, based on pGAPZA. In combination with the optimization of the fermentation conditions (i.e. pH and temperature) resulted in the maximum yield of MK-4 up to 0.24 mg/g DCW [82].

### 3.2 Polyketides

Polyketides are a class of secondary metabolites produced by bacteria, fungi, plants, and animals and the most important source of natural product-based drugs. 6-Methylsalicylic acid (6-MSA) is the first polyketide produced by *P. pastoris*. The 6-MSA biosynthetic pathway consisting of the phosphopantetheinyl transferase (PPtase) gene from *A*. *nidulans* and the 6-MSA synthase (6-MSAS) gene from *A*. *terrus* was successfully reconstituted in *P. pastoris*. After methanol induction, the production of 6-MSA was up to 2.2 g/L in 20 h in a 5 L bioreactor, which established *P. pastoris* as a promising cell factory for future industrial production of fungal polyketides [83].

Recently, *P. pastoris* was engineered for *de novo* biosynthesis of citrinin, a value-added compound. The structure and biosynthetic pathway of citrinin are more complicated than 6-MSA, serving as an excellent model compound for further investigations [84,85]. Besides the citrinin polyketide synthase gene *PksCT* (*CitS*) from *Monascus purpureus* and the phosphopantetheinyl transferase gene *NpgA* from *A*. *nidulans*, the citrinin gene cluster from *M. purpureus*, including a serine hydrolase gene *MPL1* (*CitA*), an oxygenase gene *MPL2* (*CitB*), a dehydrogenase gene *MPL4* (*CitD*), and other two intron-removed genes *MPL6* (*CitE*) and *MPL7* (*CitC*), was introduced to enable citrinin biosynthesis in *P. pastoris*. After 24 h induction with methanol, the yield of citrinin reached up to 0.6 ± 0.1 mg/L [86].

**Fig. 4:**
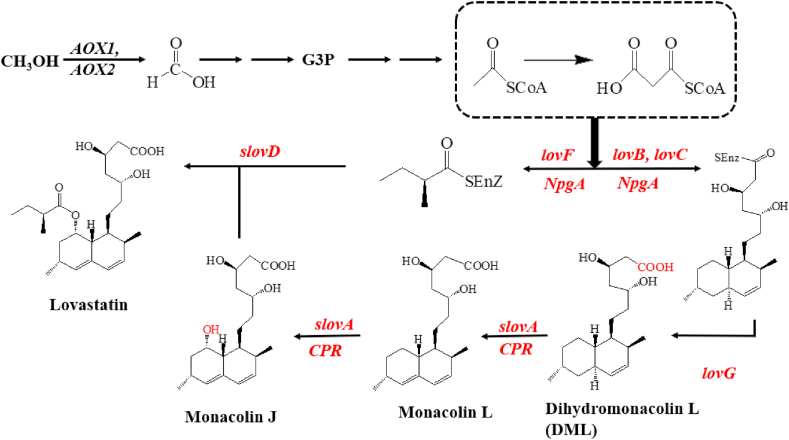
Biosynthetic pathway for lovastatin and simvastatin. Heterologous genes integrated into the genome of *P. pastoris* were shown in red. *lovB* and *lovF:* two PKS genes; *lovC:* an enoyl-reductase gene; *lovG:* a thioesterase gene; *lovA:* a cytochrome P450 monooxygenase gene; and *lovD:* an acyl-transferase gene. *NpgA* is from *A*. *nidulans*. *lovB*, *lovC*, *lovF*, *lovG,* and *CPR* were amplified from the *A*. *terreus* genome. *slovA* and *slovD* are synthetic and codon-optimized DNA sequences for *lovA* and *lovD,* respectively. (For interpretation of the references to colour in this figure legend, the reader is referred to the Web version of this article.)

Production of monacolin J and lovastatin is another classic example of the production of polyketides using *P. pastoris* (Fig. 4). Seven enzymes, including lovastatin nonaketide synthase (LovB), enoyl reductase (LovC), thioesterase (LovG), a cytochrome P450 enzyme (LovA) together with a cytochrome P450 reductase (CPR), and cyltransferase (LovD) from *A. terreus*, as well as phosphopantetheinyl transferase (PPtase or NpgA) from *A*. *nidulans*, were heterologously expressed in *P. pastoris*. The expression of all these genes were driven by *pAOX1*. *LovB*, *LovC*, *LovG*, and *NpgA* expression cassettes were cloned into the vector pPICZ B. *LovA* and *CPR* were cloned into the vector pPIC3.5K. After these two vectors were linearized and integrated into the GS115 genome, the recombinant strains were screened using zeocin-containing plates and histidine-deficient plates, respectively. Under pH-controlled culture conditions with methanol as the carbon source, 60.0 mg/L of monacolin J and 14.4 mg/L of lovastatin were obtained. In order to overcome the limitations of intermediate accumulation and metabolic burden, approaches using pathway splitting at the branch point of dihydromonacolin L and co-cultivation of yeast species were developed. Under the optimal conditions, 593.9 mg/L monacolin J and 250.8 mg/L lovastatin were obtained [18]. Compared with the yield of lovastatin in *S. cerevisiae* (20 mg/L), *P. pastoris* obviously demonstrated higher potential for further development and practical applications [87].

### 3.3 Flavonoids

**Fig. 5:**
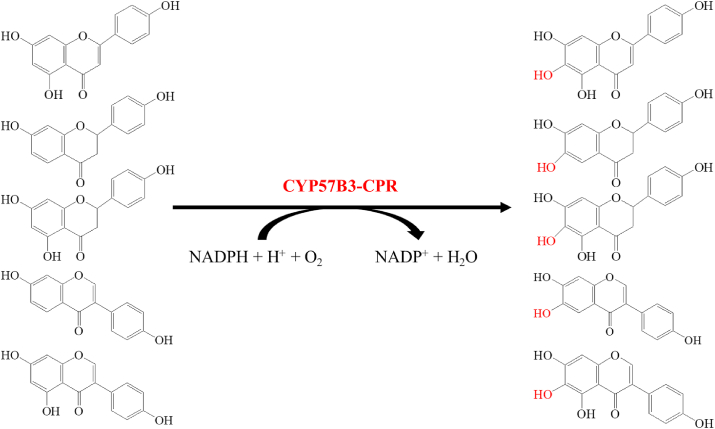
The ortho-hydroxylation of five flavonoids. CYP57B3-CPR is a fusion protein consisting of CYP57B3 from *A*. *oryzae* and the cytochrome P450 reductase (CPR) from *S. cerevisiae*.

Flavonoids are widely existed in plants and used as essential components of many drugs, such as those to prevent cardiovascular and cerebrovascular diseases and treat chronic hepatitis [88]. Chang et al. used a recombinant *P. pastoris*, which expressed a fusion protein of CYP57B3 from *A*. *oryzae* and CPR from *S. cerevisiae*, to catalyze the *ortho*-hydroxylation of 10 flavonoids. The results showed that 5 flavonoids, including genistein, daidzein, liquiritigenin, naringenin, and apigenin could be transformed into the corresponding hydroxyl derivatives (Fig. 5). In addition, after feeding with genistein, the yeast extracts showed high inhibitory activity on melanogenesis [89]. Then, 3′-hydroxygenistein was identified as the active product of genistein biotransformation in recombinant *P. pastoris*, which demonstrated liver protection and anti-inflammatory effects. The conversion yield of 3′-hydroxygenisterin was 14% with a production level of 3.5 mg/L in a 5 L fermenter [90]. Furthermore, the periodic hydrogen peroxide shock strategy was employed to further increase the production of 3′-hydroxygenistein to 20.3 mg/L in a 5 L fermenter [91].

## 4 Conclusions and perspectives

Many synthetic biology tools have been developed to precisely control the expression of heterologous genes and the assembly and integration of multi-gene pathways in *P. pastoris* [75]. However, the development of *P. pastoris* cell factories for natural products is still limited to a few examples [82], particularly when compared with that of *S. cerevisiae*, indicating a need to develop novel synthetic biology tools. For example, the most commonly used promoters in *P. pastoris* are still *pAOX1* and *pGAP* [51], while the construction of efficient cell factories generally requires to precisely control the expression levels of biosynthetic pathway genes. In this case, promoters with different strength (weak, medium, and strong promoters) should be characterized under various fermentation conditions (i.e. carbon sources). In addition, most of exogenous genes are currently integrated into the *HIS4* or *AOX1* locus of the *P. pastoris* genome, while natural product biosynthetic pathways generally contain multiple genes [72]. In other words, well characterized integration sites are highly demanded to assemble natural product biosynthetic pathways. Ideally, the integration sites should enable efficient and stable expression of heterologous genes, without affecting cell fitness. Although CRISPR/Cas9 system has been established for genome editing in *P. pastoris*, multiplex integration of long biosynthetic pathways still suffers from low efficiency and the reports on the use of CRISPR technology to construct *P. pastoris* cell factories are still rather limited. Currently, simultaneous integration of multiple genes was reported in a *ku70*-deficient *P. pastoris* strain. Unfortunately, the disruption of *KU70* was generally reported to result in impaired fitness and low transformation efficiency [72,92]. The challenge in improving homologous recombination efficiency without *KU70* disruption should be addressed for multiplex integration of natural product biosynthetic pathways in near future.

Due to the complexity of cellular metabolic network, systems level understanding is generally a prerequisite to establish efficient *P. pastoris* cell factories. Based on the genome sequencing results of *P. pastoris* DSMZ 70382 [93] and GS115 [94], genome-scale metabolic models (GEMs), PpaMBEL1254 [95], iPP668 [96], and iLC915 [97] have been established. Tomas-Gamisans et al. reconstructed and verified a new consensus model iMT1026 based on these three early reports of GEMs. New discoveries related to glycosylation, fatty acid metabolism, and cell energy were complemented to the new model. Growth rate, carbon dioxide production, arabitol production, and other parameters predicted by the model iMT1026 were consistent with the experimental results, confirming that the prediction and simulation capabilities have been improved [98]. The *P. pastoris* GEMs have been successfully implemented to identify targets to increase the production of recombinant proteins. Nocon et al. predicted 9 engineering targets based on GEMs, 5 of which significantly increased the production of cytosolic human superoxide dismutase (hSOD) [99]. Cankorur-Cetinkaya et al. used GEMs to analyze the metabolic burdens caused by heterologous protein synthesis and found that supplementation of tyrosine to the culture medium could increase the yield of human lysozyme and antibody fragment Fab-3H6 [100]. Currently, the production of natural products is still mainly limited by the low efficiency of the biosynthetic pathways, and there is a lack of examples on the application of GEMs in improving the production of natural products in *P. pastoris*. Nevertheless, once the bottleneck of the pathway enzymes has been addressed and the titer reaches to a certain level, the supply of the precursors and cofactors will become rate-limiting and GEMs can play a more important role in guiding the design of efficient yeast cell factories.

As a non-model yeast strain, our understanding of the metabolic and regulatory networks is still rather limited for *P. pastoris.* For example, Wriessnegger et al. found that the overexpression of *RAD52*, encoding a protein responsible for DNA repair and recombination, significantly improved the production of trans-nootkatol [79]. Therefore, it is highly desirable to develop genome-scale metabolic engineering strategies that can perturb all the genes at once, with an aim to identify non-intuitive engineering targets to improve the production of the desirable compounds and map genotype-phenotype relationships in a high-throughput manner [101]. Currently, CRISPR based genome-scale engineering has been well established in *E. coli* [102], *S. cerevisiae* [[103], [104], [105]], and mammalian cells [106]. Nevertheless, several challenges should be addressed to establish genome-scale engineering for *P. pastoris*, such as an efficient CRISPR system, high transformation efficiency, as well as high throughput screening.

In summary, great progress has been made in genetic manipulation (i.e. heterologous gene expression system and genome editing system) of *P. pastoris* for the production of recombinant proteins and value-added compounds. Nevertheless, more efforts should be devoted to developing novel synthetic biology tools (i.e. multi-gene pathway assembly and multiplex genome engineering) to establish *P. pastoris* as a robust chassis for the biosynthesis of natural products.

## CRediT author statement

Juncan Gao and Lihong Jiang drafted the manuscript. Jiazhang Lian conceived the review idea and revised the manuscript. All authors approved the manuscript.

## Declaration of competing interest

None.
